# Neoadjuvant programmed death ligand-1 with chemotherapy versus chemotherapy alone for limited-stage small-cell lung cancer: a retrospective study

**DOI:** 10.3389/fonc.2025.1470445

**Published:** 2025-01-30

**Authors:** Zhi Yang, Yan-qing Wang, Xiujun Chang

**Affiliations:** Department of Thoracic Surgery, Beijing Chest Hospital, Capital Medical University, Beijing, China

**Keywords:** small-cell lung cancer, immune checkpoint, neoadjuvant, tumor markers, chemotherapy

## Abstract

**Summary background:**

Our objective was to investigated the safety and feasibility of neoadjuvant treatment with PD(L)1 inhibitors and chemotherapy followed by surgery for resectable SCLC.

**Methods:**

In this retrospective cohort study, we included patients with limited-stage SCLC treated with neoadjuvant chemotherapy (with/without)ICI at Beijing Chest Hospital (Beijing, China) between July 2020 and December 2021. Seventeen patients with LD-SCLC were enrolled in the study. Two groups were assigned for further statistical analysis: neoadjuvant chemotherapy (group C), in which only preoperative chemotherapy was administered; and neoadjuvant ICI (group I), in which surgery was combined with both preoperative ICI and chemotherapy. Patient demographics, radiological and pathological evaluations of tumor response, surgical information, toxicity profiles, tumor marker and follow-up results of both groups were evaluated.

**Results:**

17 patients were included in this retrospective study, of which, 11 patients received ICI and chemotherapy-containing regimens and 6 patients received neoadjuvant chemotherapy only. Herein, we firstly reported that neoadjuvant PD-(L)1 blockade plus chemotherapy led to a pCR rate of 45.5% in patients with limited-stage small cell lung cancer. The MPR rate of 72.7% due to treatment with neoadjuvant PD-(L)1 blockade plus chemotherapy group (group I) was significantly higher than those in the traditional neoadjuvant chemotherapy group (16.7%)(group C). We first found that ProGRP is a good the evaluation indicator for neoadjuvant immunotherapy in small cell lung cancer and found that the ProGRP levels decreased significantly in both group after neoadjuvant therapy, and it was more obvious in group I(P=0.003).All Of the 17 patients (100.0%) had R0 resection. There were no perioperative deaths.

**Conclusions:**

Neoadjuvant immunotherapy shows lower toxicity and fewer perioperative complications. ICI combined chemotherapy can achieve more pathological relief and clinical benefits in the neoadjuvant treatment of LS-SCLC without increased irAE and perioperative complications. However, the small sample size limits the reliability of the research.

## Introduction

Lung cancer is the second most commonly diagnosed cancer and the leading cause of cancer-related death worldwide (1). Small-cell lung cancer (SCLC) accounts for 13%–15% of all lung cancers. It is an extremely aggressive neuroendocrine tumor characterized by early and rapid spread, and an early tendency to metastasise (2). As such, it has a very poor prognosis, with a median survival of usually approximately 15–20 months, and a two-year survival rate of 5% (3).

Staging for SCLC was initially defined by the Veterans Administration Lung Study Group (VALSG) in the 1950s as limited-stage (LS) and extensive-stage (ES) disease; LS-SCLC is a disease confined to one hemithorax that can be safely encompassed in a single radiation portal (4). In 1987, the International Association for the Study of Lung Cancer revised the VALSG system to adapt it to the TNM staging system, with LS disease including stages I to III and ES disease including stage IV (5). The National Comprehensive Cancer Network guidelines already recommend surgical treatment for patients with very limited disease (clinical T1–2, N0) (6). However, there is no consensus regarding the surgical approach for more advanced, operable stages. In recent years, some studies have altered the status of surgery in patients with this disease. These studies have shown that patients with early stage SCLC may benefit from surgery (7–9). In 2017, Yang et al. (10) compared patients with N1 disease who underwent surgical resection with adjuvant chemotherapy versus (vs.) concurrent chemoradiation alone. Surgery plus chemotherapy was associated with improved overall survival (hazard ratio [HR] 0.74 [95% confidence interval (CI) 0.56–0.97]) and five-year survival (31.4% vs. 26.3%; P=0.03). In 2014, Xu et al. (11) found that neoadjuvant chemotherapy combined with surgery provided reasonable options for patients with pIIIa-N2 LS-SCLC, which could increase their chances of survival. Despite recent advances, these findings highlight the need for new and more effective multimodal treatment strategies to improve the long-term survival of patients with LS-SCLC.

Immune checkpoint inhibitors (ICIs) have emerged in the past few years and have revolutionized the treatment of non-small cell lung cancer (NSCLC) and other advanced solid malignancies. Anti-programmed death ligand-1 (PD-L1) and anti-programmed death protein 1 (PD-1) antibody immunotherapy has already been used in the treatment of metastatic SCLC and has yielded encouraging therapeutic results (12). Atezolizumab is a recombinant humanized anti-PD-L1 monoclonal antibody that blocks interactions between PD-1 and its ligands, and previous clinical trials have reported that Atezolizumab achieved a good effect in ES-SCLC with few side effects (13). Recently, a phase III clinical trial evaluating the efficacy and safety of tislelizumab for ES-SCLC (RATIONALE-312) updated its findings at the 2023 WCLC meeting. The median overall survival (OS) for first-line treatment of ES-SCLC with tislelizumab and EP was 15.5 months (vs. 13.5 months of chemotherapy; HR 0.75, P=0.0035), and the median progression-free survival (PFS) was 4.8 months (vs. 4.3 months of chemotherapy). Nevertheless, ICIs are not widely used in the neoadjuvant phase, and the literature addressing neoadjuvant immunotherapy for SCLC is sparse, with only a few case reports and case series alluding to neoadjuvant immune treatment for SCLC (14). This indicates a lack of research investigating neoadjuvant immunotherapy for SCLC. As such, the present study investigated the safety and feasibility of neoadjuvant treatment with PD(L)1 inhibitors and chemotherapy followed by surgery for resectable SCLC.

## Patients and methods

### Patients

The clinical records of all patients who underwent neoadjuvant chemotherapy (with/without) ICI for limited-stage SCLC (stages I-IIIA according to the American Joint Committee on Cancer, 8th Edition lung cancer staging system), followed by radical surgical resection at Beijing Chest Hospital (Beijing, China) between July 2020 and December 2021, were retrospectively reviewed. Seventeen patients with LD-SCLC were enrolled in the study. Two groups were assigned for further statistical analysis: neoadjuvant chemotherapy (group C), in which only preoperative chemotherapy was administered; and neoadjuvant ICI (group I), in which surgery was combined with both preoperative ICI and chemotherapy. During assessment before treatment, all patients underwent a detailed interview, physical examination, chest computed tomography (CT), head magnetic resonance imaging (MRI) or CT, electrocardiography, pulmonary function tests, abdominal ultrasound, whole-body bone scan, and bronchoscopy. The ethics committee of the hospital approved the retrospective collection of data and analysis, and written informed consent was obtained from all patients (Ethics Approval No. BJXK-2021-KY-06).

Patients underwent neoadjuvant chemotherapy (platinum plus etoposide) combined with atezolizumab (Roche Pharmaceutical Co., Ltd., Basel, Switzerland) or tislelizumab (BeiGene, Ltd., Beijing, China) for two to three cycles at an interval of three weeks before surgery. Platinum-based chemotherapy consisted of cisplatin (75 mg/m^2^), carboplatin (area under the receiver operating characteristic [ROC] curve for drug plasma concentration [AUC] = 5), or lobaplatin (50 mg/m^2^) and etoposide (60–100 mg/m^2^). After neoadjuvant therapy, tumor stage was re-evaluated using chest CT, abdominal ultrasound, brain MRI, whole-body bone scan, and bronchoscopy. Adjuvant therapy was adjusted according to pathological stage using the same drugs. ICI was continued for almost 1 year for patients who complied with medical advice.

### Data collection and evaluation

The following demographic information and patient characteristics were collected from the institutional database: sex; age; surgery-related details and complications, toxicity profiles, and prognostic outcomes; comorbidity; history of smoking; neoadjuvant and adjuvant chemotherapy (timing and dosage); neoadjuvant and adjuvant ICI (timing and dosage); histology; tumor location; pathological T, N, and TNM stage, operative duration (min); blood loss volume (mL); R0 surgery rate; complications such as bleeding, acute respiratory distress syndrome (ARDS), bronchopleural fistula, atrial fibrillation, esophageal injury, and chylothorax. Survival status was obtained from clinical medical records or telephone follow-up(s). Radiological response was assessed according to the Response Evaluation Criteria in Solid Tumors (RECIST) version 1.1 (15). Radiological responses were classified as complete response (CR, no residual disease), partial response (PR, no less than 30% reduction in size), progressive disease (PD, no less than 20% increase in size or occurrence of new lesions), and stable disease (SD, < 20% increase and < 30% reduction in size).

Routine haematoxylin and eosin (H&E) staining was used to identify the percentage of residual viable tumors from the primary tumors. Pathological CR (pCR) and major pathological response (MPR) were considered as 0% and ≤ 10% of viable tumor cells remaining in residual tumor, respectively. Toxicity profiles, including treatment-related adverse events (AEs) and abnormal laboratory findings, were graded using the National Cancer Institute Common Terminology Criteria for Adverse Events, version 5.0.

### Follow-up

Follow-up was performed during outpatient visits or through telephone calls. The final follow-up visit was in June 2023.

### Statistical analysis

Descriptive analyses of patient demographics, radiological and pathological evaluations of tumor response, surgical information, toxicity profiles, and follow-up results were performed, and demographic and disease-specific variables were compared using the chi-squared test for categorical variables and the *t*-test for continuous variables. The event-free survival analysis was performed using the Kaplan–Meier method and log-rank test. Differences with P < 0.05 were considered to be statically significant. Statistical analysis was performed using SPSS version 19.0 (IBM Corp, Armonk, NY, USA) for Windows (Microsoft Corporation, Redmond, WA, USA) and R studio, version 3.6.3 (R Foundation for Statistical Computing, Vienna, Austria).

## Results

### Patient characteristics

Seventeen patients with LS-SCLC underwent neoadjuvant chemotherapy with or without ICI, followed by radical surgical resection, between July 2020 and December 2021. Demographic characteristics of the 17 patients are summarized in **Table 1**. In addition to radical resection, 11 patients underwent neoadjuvant ICI and chemotherapy (group I), and six underwent neoadjuvant chemotherapy with etoposide and platinum (group C).

**Table 1:** Clinical characteristics.

Clinical Characteristics	Type of neoadjuvant treatment			p-value
	Group I (N=11)	Group C (N=6)		
Sex				
Male	9(81.8)	5(83.3)		0.938
Female	2(18.2)	1(16.7)		0.938
Age,y	58(43-69)	56(45-65)		0.657
Smoking				0.627
Never	5(45.5)	2(33.3)		
Current(Yes/Ever)	6(54.5)	4(66.7)		
Drug				
atezolizumab	6(54.5)	0		
tislelizumab	5(45.5)	0		
Surgery				0.901
VATS	4(36.4)	2(33.3)		
Open	7(63.6)	4(66.7)		
Surgery				
Mean operative time, min	134.5	133.3		0.956
Mean estimated blood loss, ml	195.5	66.7		0.078
Surgical margin , R0	11(100)	6(100)		
Surgery				0.596
Lobectomy	5(45.5)	5(83.3)		
Bilobectomy	5(45.5)	0		
Pneumonectomy	1(9.1)	1(16.7)		
Postoperative pathology				0.027
CPR	6(54.5)	1(16.7)		
MPR	8(72.7)	1(16.7)		

Data are presented as mean ± SD, median (P25–P75) or n (%). BMI, body mass index; LUL, left upper lobectomy; LLL, left lower lobectomy; PD-L1, Programmed death-ligand 1; RUL, right upper lobectomy; RLL, right lower lobectomy; VATS, video-assisted thoracoscopic surgery; CPR, complete pathological response; MPR, major pathological response.

The mean age of patients in group I (nine male [81.8%], two female [18.2%]) at the time of surgical resection was 58 years, of whom six (54.5%) had a history of smoking (**Table 1**). Pre-chemotherapy clinical staging among these 11 patients was as follows: stage Ia (n=1 [9.2%]); stage IIb (n=2 [18.2%]); stage IIIa (n=6 [54.5%]); and stage IIIb (n=2 [18.2%]). Restaging after chemoimmunotherapy was as follows: stage Ia (n=2 [18.2%]); stage Ib (n=1 [9.1%]); stage IIb (n=3 [27.3%]); and stage IIIa (n=5 [45.5%]) (**Table 2**).

**Table 2A:** Stages of group I.

Patient	Pre-chemotherapy	Restaging	Postoperative	CPR	MPR	PPR or SD
1	c-t2an1------2b	T1bn1--2b	Occult (TxN0M0)	+		
2	c-t2an1------2b	T1cn1—2b	t1n0----1a			+
3	c-t3n1-------3a	T1bn0—1a	Occult (TxN0M0)	+		
4	c-t3n1------3a	T2an0---1b	t1an1----2b		+	
5	c-t3n2------3b	T1bn2--3a	t1an0----1a		+	
6	c-t1cn0-----1a	T1an0--1a	Occult (TxN0M0)	+		
7	c-t3n1-----3a	T1bn1--2b	Occult (TxN0M0)	+		
8	c-t2an2----3a	T1bn2--3a	Occult (TxN0M0)	+		
9	c-t2an2----3a	t1bn2--3a	t1n2----3a			+
10	c-t3n2----3b	t1an2--3a	Occult (TxN0M0)	+		
11	c-t1bn2----3a	t1bn2--3a	t1n2----3a			+

CPR, complete pathological response; MPR, major pathological response; PPR partial pathological response; SCC: squamous cell carcinoma; Tx, cancer cells were not found pathologically.

**Table 2B:** Stages of group C.

Patient	Pre-chemotherapy	Restaging	Postoperative	CPR	MPR	PPR or SD
1	c-t2an0------1b	T1an0—1a	Occult (TxN0M0)	+		
2	c-t1bn1------2b	T1bn1—2b	t1bn0			+
3	c-t1cn2-------3a	T1cn2—3a	t1cn2			+
4	c-t1cn0------1a	T1bn0---1a	t1bn0			+
5	c-t2n2------3a	T1cn2--3a	t1cn1			+
6	c-t2n2-----3a	T1bn2--3a	t1cn2			+

CPR, complete pathological response; MPR, major pathological response; PPR partial pathological response; SCC: squamous cell carcinoma; Tx, cancer cells were not found pathologically.

The mean age of patients in group C (five male [83.3%], one female [13.9%]) at the time of surgical resection was 56 years, of whom four (66.7%) had a history of smoking. Pre-chemotherapy clinical staging was as follows: stage Ia (n=1 [16.7%]); stage Ib (n=1 [16.7%]); stage IIb (n=1 [16.7%]); and stage IIIa (n=3 [50%]). Restaging after chemotherapy was as follows: stage Ia (n=2 [33.3%]); stage IIb (n=1 [16.7%]); and stage IIIa (n=3 [50%]).

All patients underwent radical pulmonary resection (R0) and radical lymphadenectomy according to the American Thoracic Society classification, removing all lymphatic tissue from stations 2R, 4R, 7, and 10R for right-sided tumors, and from stations 5, 6, 7, and 10 L for left-sided tumors. All 17 patients had preoperative SCLC, of whom 12 underwent bronchoscopic brush biopsy or bronchoscopic biopsy, three underwent transthoracic needle pneumocentesis, and two underwent endobronchial ultrasound.

### Treatment regimens and responses

#### Assessment

**Figure 1:**
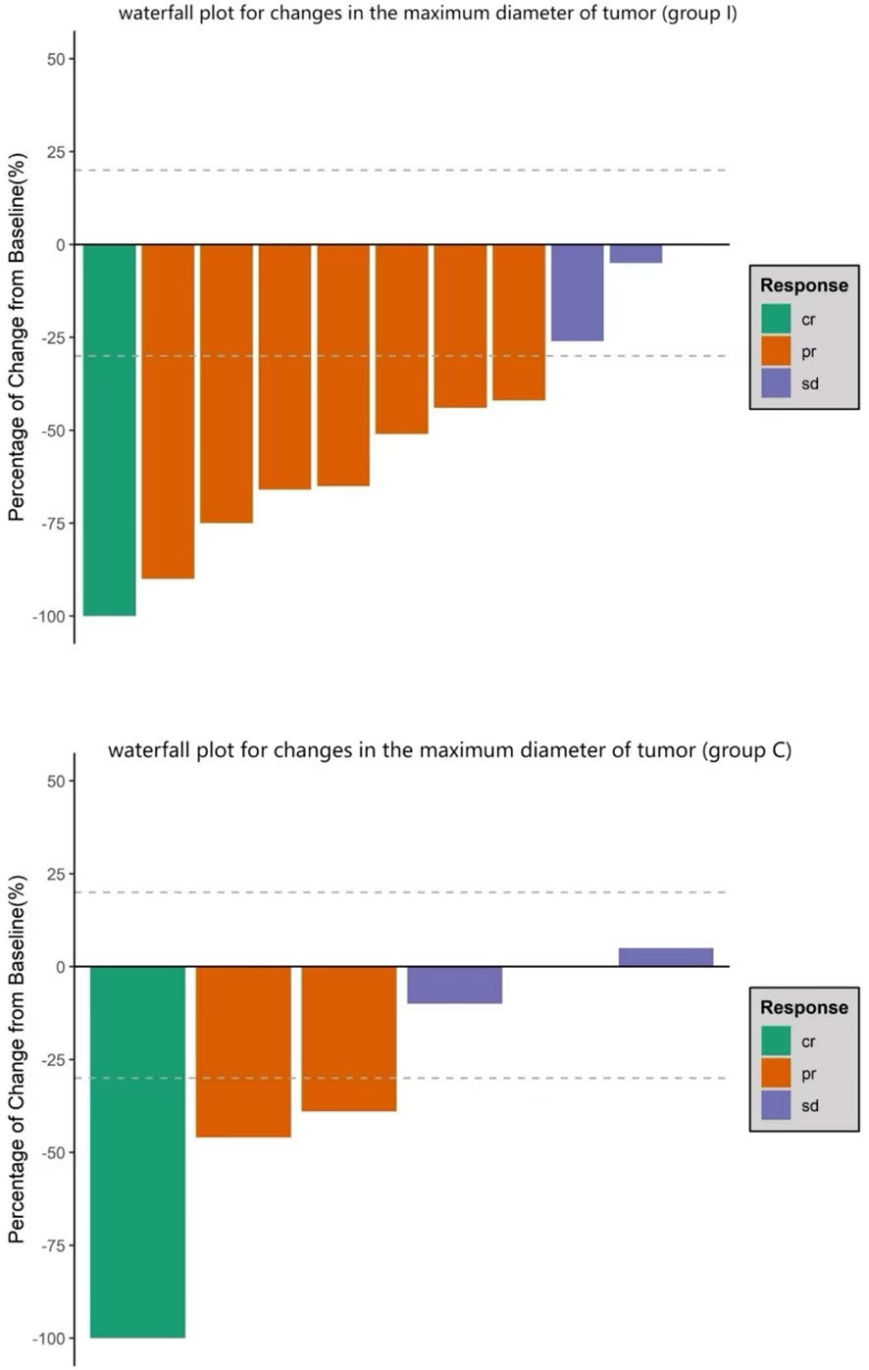
Waterfall plot for changes in the maximum tumor diameter. Bars represent data from individual patients. Negative values suggest tumor shrinkage and positive values suggest progressive disease; the dashed lines represent the thresholds for a partial response (shrinkage by 30%) or for progressive disease (growth by 20%) according to the Response Evaluation Criteria in Solid Tumors (i.e., “RECIST”) criteria.

**Figure 2:**
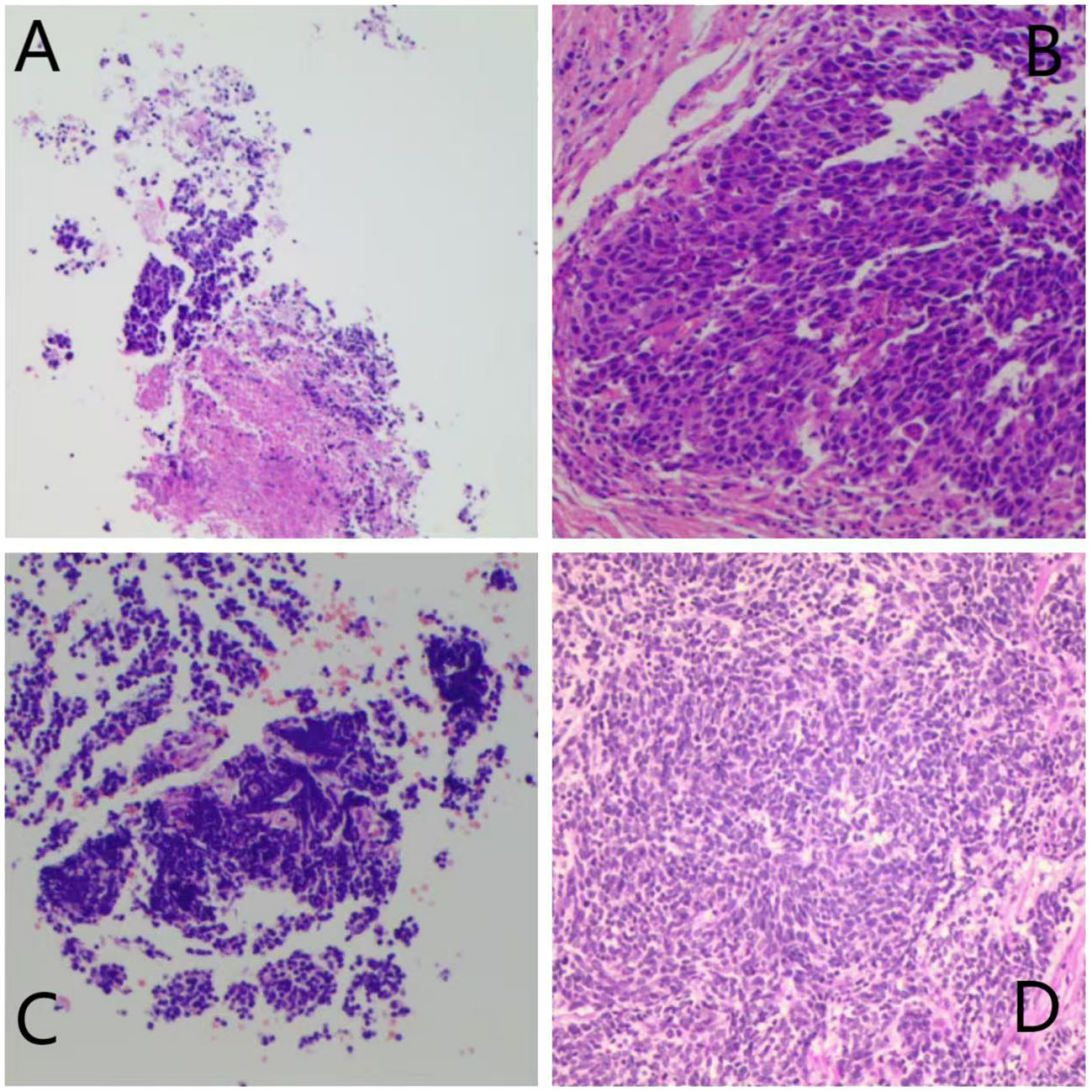
Preoperation **(A)** and postoperation **(B)** pathological HE staining results of Patient 4 in Group I.Major pathological response was observed in this patient. Preoperation **(C)** and postoperation **(D)** pathological HE staining results of Patient 2 in Group C.

The treatment regimens for all patients were determined jointly by the treating surgeons and oncologists through multidisciplinary discussion. In group I, six patients were treated with atezolizumab (Roche Pharmaceutical Co., Ltd., Basel, Switzerland) combined with platinum and etoposide as neoadjuvant treatment; the other patients received tislelizumab (BeiGene, Ltd., Beijing, China) and the same chemotherapy regimen. Preoperative chemotherapy combined with ICI was administered for two to three cycles. In group I, the median interval between the last administration of ICI and surgery was 39 days (range, 26–52 days). Preoperative CT evaluation in group I revealed CR in one patient and PR in seven, with an objective response rate (ORR) of 72.7% (**Figure 1**). Regarding pathological response, six had a complete pathological response (CPR) (54.5%) and eight had MPR (72.7%) (**Table 2A**). The MPR rate was 66.6% (4/6) in six patients who underwent atezolizumab immunotherapy and 80% (4/5) in five who underwent tislelizumab immunotherapy. There were no statistically significant differences between the groups (P=0.576). Postoperative pathology usually reveals hyperplasia of the bronchial and peribronchial fibrous tissues with transparent degeneration, necrosis, tumour-infiltrating lymphocytes (TILs), proliferative fibrosis, and a small amount of residual tumor (**Figure 2**). Postoperative pathological evaluation revealed pathological downstaging in nine of the 11 (81.8%) patients (**Table 2**).

In group C, six patients were treated with platinum and etoposide as neoadjuvant treatment regimens. The median interval between the last dose of chemotherapy and surgery was 32 days (range, 24–55 days). Postoperative pathology revealed that one patient (16.7%) exhibited CPR (**Table 2B**). The MPR rates in the ICI and chemotherapy combined with neoadjuvant therapy group (72.7%) were significantly higher than those in the neoadjuvant chemotherapy group (16.7%) (P=0.027).

#### Toxicity profile

In group I, seven patients experienced treatment-related AEs during neoadjuvant therapy. No AEs of grade ≥ 4 were observed. Five had myelosuppression, one had diarrhea, and one exhibited increased aminotransferase levels. The myelosuppressed patient was treated with antibiotics and granulocyte colony-stimulating factors. All patients recovered well. The patient with diarrhea was treated with fluids and chemotherapy was continued. The patient recovered completely and underwent surgery 26 days later. None of the patients experienced drug withdrawal or surgical delays owing to AEs.

#### Surgery-related information

All patients underwent lobectomy or pneumonectomy and systemic lymph node dissection, with seven (63.6%) undergoing open thoracotomy and four (36.4%) undergoing video-assisted thoracoscopic surgery (VATS) (**Table 1**) in group I and four (66.7%) undergoing open thoracotomy and two (33.3%) undergoing VATS in group C. None of the patients who underwent VATS experienced intraoperative conversion to open thoracotomy. In group I, lobectomy was performed in five patients: right upper lobectomy (n=2), right lower lobectomy (n=1), left upper lobectomy (n=1), and left lower lobectomy (n=1). Bilobectomies were performed in five patients, including right middle and upper lobectomy (n=1) and right middle and lower lobectomy (n=4). Left pneumonectomy was performed in one patient. All patients underwent complete tumor resection. The mean operative duration was 134.5 min, and the mean bleeding volume was 195.5 ml. No perioperative deaths occurred. Post-induction complications are reported in **Table 3**. Three (27.3%) patients experienced postoperative complications. One patient who underwent left lower lobe lobectomy experienced a postoperative air leak for seven days, which improved with conservative treatment. One patient experienced wound healing problems three months after surgery and was cured by debridement and suture surgery. Another patient developed chylothorax on postoperative day 1, which was cured by thoracic duct ligation on postoperative day 14.

**Table 3:** Postinduction therapy complications.

Patient	Complication	Time	Treatment	Outcomes
4	Myelosuppression After 2nd dose of	After 2nd dose of chemotherapy	Antibiotics and granulocyte colony stimulating factors	Complete recovery
7	Myelosuppression After 1nd dose of			
3	Increased aminotransferases			

In group C, four (66.7%) patients underwent open thoracotomy and two (33.3%) underwent VATS. Lobectomy was performed in five patients, including right lower lobectomy (n=1), left upper lobectomy (n=2) and left lower lobectomy (n=2). Right pneumonectomy was performed in one patient. All patients underwent complete tumor resection. The mean operative duration was 133.3 min, and the mean bleeding volume was 67.7 ml. There was no statistical difference between the two groups in terms of operative duration or intraoperative bleeding volume. One patient experienced postoperative complications and prolonged air leaks with prolonged chest tube drainage under negative pressure (>2 weeks).

#### Tumour marker testing

**Figure 3:**
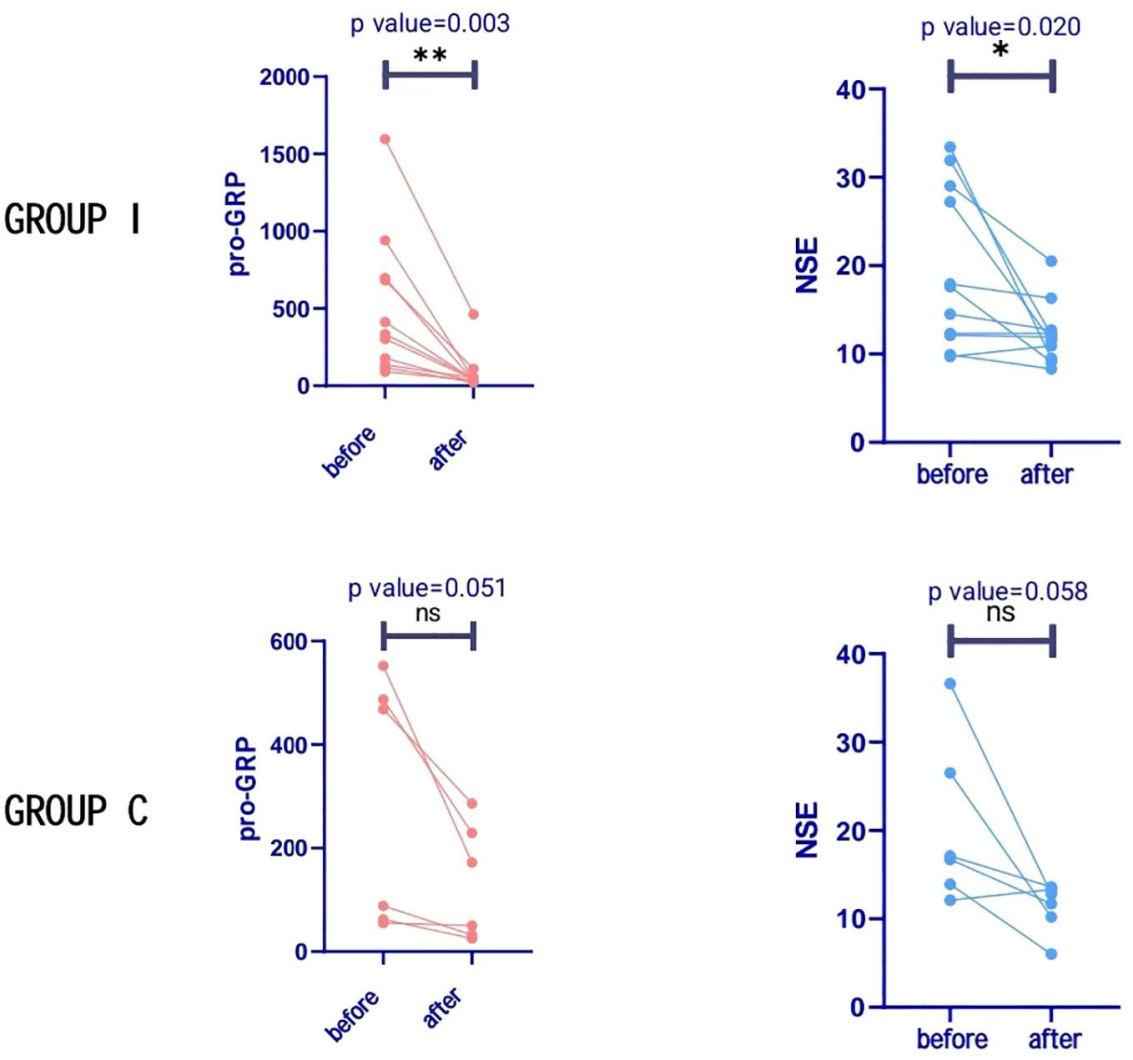
Tumour markers in both groups. Progastrin-releasing peptide (ProGRP) levels decreased significantly in group I after treatment, but with no significant difference in group C. Neuron-specific enolase (NSE) levels were significantly reduced in group I after treatment, but with no significant difference in group C *P < 0.05; **P < 0.01 versus before treatment; NS not significant.

Pretreatment and preoperative pro-gastrin-releasing peptide (ProGRP) and neuron-specific enolase (NSE) levels were continuously monitored. Preoperative ProGRP was more closely associated with treatment effectiveness than NSE. Successively, ProGRP and NSE were categorized and defined to be positive according to the following criteria: cut-off > 80 pg/mL for ProGRP; > 40 ng/mL for NSE. In group I, all patients’ ProGRP level exhibited a significant decrease after neoadjuvant therapy, and only two patients had a ProGRP level higher than normal (80 ng/mL) after treatment. This was not as evident for NSE. ProGRP level did not fall to normal level after treatment in two patients and, intriguingly, both developed early metastases (6 months and 10 months) after surgery and were the only two to develop metastases during the follow-up period. Changes in ProGRP and NSE levels in patients are presented in **Figure 3**. In group I, the preoperative ProGRP level has significantly decreased compared to pre-treatment (mean 498.7 vs. 82.3 pg/mL; P=0.003), while relatively slightly decreased NSE levels (19.6 vs. 12.3 ng/mL) were found (P=0.020). In group C, the preoperative ProGRP level also decreased compared with the pretreatment (mean 285.3 vs. 111.7 pg/mL); however, the difference was not statistically significant (P=0.051), while slightly reduced NSE levels (20.5 vs. 11.3 ng/mL) were found (P=0.058).

#### Outcomes of event-free survival

**Figure 4:**
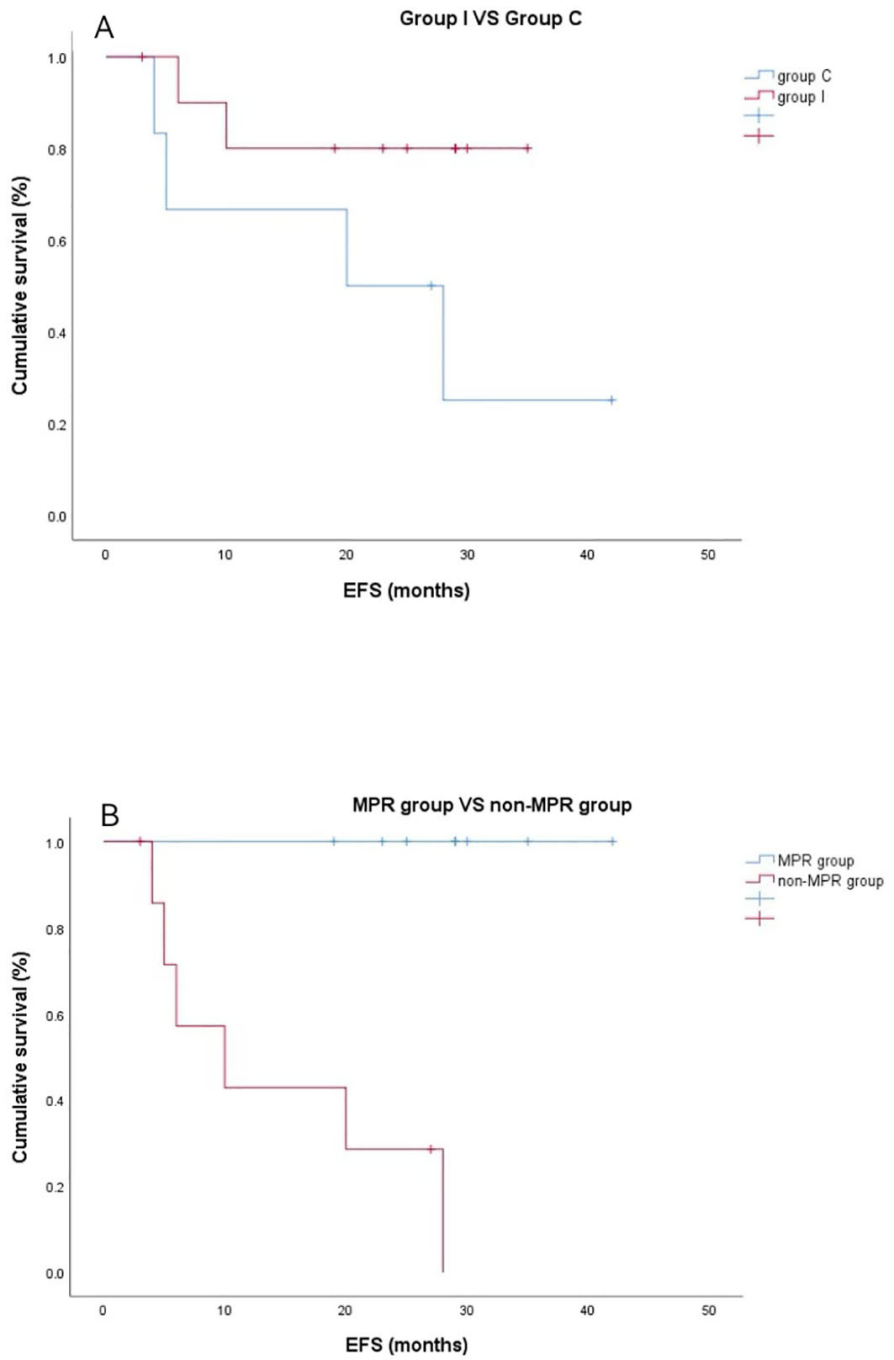
Kaplan–Meier event-free survival curves. **(A)** Neoadjuvant programmed death ligand-1 (PD-[L]1) blockade plus chemotherapy group versus neoadjuvant chemotherapy group (P=0.073). **(B)** The major pathological response (MPR) group exhibited superior recurrence-free probability compared with the non-MPR group (*P < 0.001).

One patient was lost to follow-up; regular postoperative follow-ups were performed in the remaining 16. The median follow-up was 24.0 months (range, 12–42 months). At the end of the follow-up, one patient in group C died of respiratory failure due to pulmonary infection, and metastasis and recurrence were found in five patients, including two in group I (bone metastases in both) and three in group C (one each of pericardial metastasis, adrenal metastasis, and pleural metastasis). Postoperative pathology in all five patients who developed metastases did not achieve MPR or PCR. Survival analysis was performed using the Kaplan–Meier method to compare the survival differences between patients in the two groups. Average event-free survival (EFS) time in group I was 29.6 months (95% CI 26.9–36.3 months) and 22.3 months (95% CI 10.4–34.3 months) in group C. There was no significant difference in EFS (P = 0.073) between the two groups (**Figure 4A**). Meanwhile, the median EFS for non-MPR patients was 8.0 months, whereas the median EFS for MPR patients was 29.0 months (P < 0.001) (**Figure 4B**).

## Discussion

With the gradual introduction of ICI into clinical applications, they have played a role in the treatment of various types of cancer and are effective in the treatment of ES-SCLC. The Impower133 study suggested that Atezolizumab combined with chemotherapy can improve the survival time of patients with ES-SCLC, with a median OS extension of 2 months compared with chemotherapy alone (12.3 months vs. 10.3 months) (13). Recently, the 2023 WCLC conference report showed that the combination of tislelizumab and chemotherapy also demonstrated good therapeutic effects in patients with ES-SCLC, with one-year PFS of 20.7%, which was four times higher than that in the control group (4.5%) (16). Considering the significant survival benefits achieved by first-line PD-(L)1 blockade and chemotherapy in ES-SCLC (13, 17), data regarding neoadjuvant treatment are important and anticipated in LS-SCLC.

Currently, only a few case reports and case series have addressed neoadjuvant immunotherapy plus chemotherapy for LS-SCLC. However, these studies had small sample sizes and reported inconsistent results. One case report (14) suggested that neoadjuvant chemoimmunotherapy achieved the results of postoperative pCR in stage IIB LS-SCLC. However, in a study involving patients with stage IIIA-IIIB LS-SCLC, Meng et al. (18) reported that no patient achieved postoperative pathological MPR or pCR. Liu et al. (19) reported that the rates of pCR and MPR were 30.0% and 40.0%, respectively, in patients with stage I–IIIA SCLC who received neoadjuvant immunotherapy combined with chemotherapy. In an analysis of the role of chemotherapy in combination with atezolizumab as neoadjuvant therapy in patients with resectable SCLC by Duan et al. (20), the pCR and MPR in the intention-to-treat (ITT) cohort were 47.1% and 70.6%, respectively, and most patients (90%–100.0%) underwent R0 resection after PD-(L)1 blockade-based neoadjuvant treatment combined with chemotherapy. In our study, we tentatively administered neoadjuvant PD-(L)1 blockade plus chemotherapy to 11 patients with LS-SCLC (i.e., group I) and administered neoadjuvant chemotherapy alone to six patients (i.e., group C) as controls. Pathologically, six (54.5%) patients achieved pCR, eight (72.7%) achieved MPR in group I, and one (16.7%) achieved pCR in group C. Compared with neoadjuvant chemotherapy alone, the addition of PD-(L)1 blockade significantly improved the MPR rate (72.7% vs. 16.7%; P=0.027). The results of our study were consistent with recently reported outcomes (20), indicating the satisfactory performance of neoadjuvant PD-(L)1 blockade-based immunotherapy combined with chemotherapy in optimizing the outcomes of patients with LS-SCLC.

Major pathological response to neoadjuvant treatment is a potential surrogate endpoint of survival (21). This could shorten the time required to evaluate neoadjuvant therapies and provide a faster means of comparing different neoadjuvant treatment regimens. In previously published trials investigating NSCLC, MPR rates were reported to be as high as 57% for patients in the neoadjuvant ICI and chemotherapy cohorts (22), much higher than the 16% in the chemotherapy-alone group. To date, no published studies have compared the pathological responses to neoadjuvant immunotherapy plus chemotherapy and neoadjuvant chemotherapy for LS-SCLC. The MPR rate in our study, the first in the literature for this type of treated patients, was 72.7%, comparable with previous trials ranging between 0% and 70.6% (20), which is significantly higher than the 16.7% rate after neoadjuvant chemotherapy.

Many of the current immunotherapy studies investigating SCLC mostly use PD-L1 drugs, such as Atezolizumab, as immunological agents; however, we found that the MPR results in the tislelizumab group were not lower than the results of MPR in the Atezolizumab group, and there was a significant economic advantage. In addition, we found that patients in the neoadjuvant immunochemotherapy group exhibited better recurrence-free survival (RFS) results than those in the chemotherapy alone group. Despite the P value (0.073 [i.e., > 0.05]), there were differences in trends and the average RFS time in group I was 29.6 months, which suggests that there was moderate heterogeneity between the two subgroups. Statistical analysis revealed that patients with MPR gained significantly higher RFS benefits than non-MPR patients. However, the number of cases included in this study was small and further experimental confirmation is required.

Regarding safety, two patients in group I experienced preoperative complications: patient 1 experienced mild complications with grade I nausea, and patient 4 experienced grade IV hepatic damage. Only two patients developed pulmonary infection and shortness of breath after surgery. Regarding intraoperative risk, the average intraoperative bleeding volume was 195 ml, and the mean operative duration was 134.5 min in group I. There was no significant increase in intraoperative bleeding in groups I and C, and there was no significant delay in operative duration. Therefore, post-ICI surgery is considered to be safe and efficient. This is consistent with the results of neoadjuvant immunotherapy for NSCLC (23). However, the number of preoperative chemotherapy cycles varied, and further prospective studies are required to define the optimal duration of preoperative PD(L)1 therapy.

RECIST version 1.1 has been commonly applied in the radiological evaluation of tumor response after neoadjuvant treatment in various solid tumors; however, its validity in lung cancer is not optimal. We found that radiological results were frequently inconsistent with pathological results. In our study, one patient (9.1%) achieved CR and seven (63.6%) achieved PR in group I according to post-neoadjuvant radiological evaluation, while two of three patients with stable radiological evaluation achieved pCR or MPR. Therefore, it is essential to develop a method to identify patients who are most likely to respond to immunotherapy. However, indicators for the therapeutic evaluation of neoadjuvant chemoimmunotherapy for SCLC remain unclear. Currently, the common clinical tumor markers for SCLC are NSE and ProGRP. Although several studies have revealed that ProGRP can be a useful tool for determining prognosis during SCLC treatment (24, 25), the results did not involve the neoadjuvant therapy phase. Therefore, it is necessary to investigate NSE and ProGRP levels to evaluate the efficacy of neoadjuvant PD-(L)1 blockade plus chemotherapy in patients with LS-SCLC. In the present study, we continuously monitored NSE and proGRP levels during neoadjuvant chemoimmunotherapy in patients with LS-SCLC and found that ProGRP levels decreased significantly in both groups after neoadjuvant therapy, and this decrease was more obvious in group I (P=0.003). Regarding NSE, although the levels of this indicator also obviously decreased, the decrease was not significant (P=0.020). Both patients who developed early metastases after surgery had high post-treatment proGRP levels. Therefore, proGRP is more valuable than NSE in evaluating the effect of neoadjuvant chemoimmunotherapy for SCLC.

In our study, when patients who received neoadjuvant PD-(L)1 blockade plus chemotherapy achieved MPR or pCR, ProGRP levels decreased to normal before surgery (8/8), while patients with higher-than-normal preoperative ProGRP levels did not achieve MPR or pCR (2/2). To confirm the predictive value of increased ProGRP levels, we further analyzed the RFS of these patients and found that the tumor recurrence rate of patients with higher-than-normal ProGRP levels before surgery was significantly higher than that of patients with normal levels. Elevated ProGRP levels are associated with poor outcomes of neoadjuvant chemoimmunotherapy and tumor recurrence and may help predict prognosis and screen patients more suitable for PD-(L)1 blockade immunotherapy-based neoadjuvant treatment in LS-SCLC. However, these outcomes should be further verified in adequately powered studies.

The present study had several limitations, the first of which was its single-center, retrospective design; therefore, selection bias may have occurred. Second, the sample size was small and the follow-up period was short. Larger studies are needed to correlate the pathological response to neoadjuvant therapy with OS. Third, we used two different ICIs. Finally, our results may have been influenced by tumor characteristics and the surgeons’ technique, experience, and preferences.

In summary, we report 11 cases involving the use of PD-(L)1 in the neoadjuvant setting in LS-NSLC. All 11 patients exhibited promising responses to induction treatment, which enabled complete resection. In addition, good tolerance to neoadjuvant PD(L)1 was observed in all patients. In addition, we found that ProGRP was a good observational indicator of the efficacy of neoadjuvant chemotherapy in SCLC. However, this was a retrospective study with a small sample size, and the rate of response to neoadjuvant PD-(L) 1 for LS-SCLC remains unclear. If the response rate is unsatisfactory and patients experience disease progression, the chances of undergoing an operation at a relatively early stage would probably be terminated. Moreover, whether the duration of PD-(L)1 therapy improves patient OS remains to be explored. Future prospective trials focusing on neoadjuvant immunotherapy to provide solid evidence and help inform the optimal management of neoadjuvant treatment in patients with LS-SCLC are warranted.

## Data Availability

The raw data supporting the conclusions of this article will be made available by the authors, without undue reservation.
